# Effect of Diode Laser Photobiomodulation on the Stability of Orthodontic Mini-Implants: A Systematic Review of Randomized Clinical Trials

**DOI:** 10.3390/jcm15145628

**Published:** 2026-07-17

**Authors:** João Barreto-Santos, Primavera Sousa-Santos, Pedro Otão

**Affiliations:** 1 Independent Researcher, 8200-434 Albufeira, Portugal; 2 Oral Pathology and Rehabilitation Research Unit (UNIPRO), University Institute of Health Sciences (IUCS) CESPU, Rua Central da Gandra 1317, 4585-115 Gandra, Portugal; primavera.santos@iucs.cespu.pt; 3 Faculty of Dental Medicine, University of Lisbon, Rua Professora Teresa Albrósio, Cidade Universitária, 1600-277 Lisboa, Portugal; pedrootao@edu.ulisboa.pt

**Keywords:** photobiomodulation therapy, low-level light therapy, diode lasers, orthodontic anchorage procedures, implant stability, orthodontics

## Abstract

**Background/Objectives**: Orthodontic mini-implants have become an essential source of temporary skeletal anchorage because of their versatility, minimal invasiveness, and reduced dependence on patient compliance. However, their clinical success relies on maintaining adequate primary and secondary stability throughout orthodontic treatment. Photobiomodulation (PBM) with diode lasers has been proposed as a non-invasive adjunctive therapy capable of enhancing bone healing and remodeling, thereby improving mini-implant stability. Despite encouraging findings, the available evidence remains limited and heterogeneous. The objective was to systematically evaluate the effect of diode laser photobiomodulation on the stability of orthodontic mini-implants. **Methods**: A systematic review was conducted according to the Preferred Reporting Items for Systematic Reviews and Meta-Analyses (PRISMA) guidelines. The search was conducted in the PubMed, EBSCO, Scopus, and ScienceDirect databases to identify randomized clinical trials published in the last 10 years. Five randomized controlled trials involving 89 participants and 178 orthodontic mini-implants fulfilled the eligibility criteria. Mini-implant stability was assessed using objective methods, including resonance frequency analysis (ISQ) and Periotest values, while one study also evaluated insertion and removal torque. The methodological quality of the included studies was assessed using the Cochrane Risk of Bias 2 (RoB 2) tool, and the certainty of the evidence was evaluated using the GRADE approach. **Results**: Three of the five included studies reported improvements in orthodontic mini-implant stability following diode laser photobiomodulation, whereas two studies found no significant differences compared with the control group. Considerable heterogeneity was identified regarding laser wavelength (618–940 nm), irradiation protocols, energy parameters, timing of force application, and stability assessment methods. Most studies presented a low risk of bias, although some concerns remained regarding allocation concealment and blinding. According to the GRADE assessment, the overall certainty of the evidence was considered moderate, mainly because of inconsistency across studies and methodological heterogeneity. **Conclusions**: Current evidence suggests that diode laser photobiomodulation may improve the stability of orthodontic mini-implants. Nevertheless, the small number of randomized clinical trials and the substantial variability in PBM protocols and outcome assessment methods preclude definitive clinical recommendations. Future well-designed randomized controlled trials using standardized irradiation parameters and uniform stability assessment methods are required to establish evidence-based clinical protocols.

## 1. Introduction

The use of orthodontic mini-implants has become increasingly prevalent in contemporary orthodontic practice, providing enhanced anchorage control, improved biomechanical efficiency, and reduced dependence on patient compliance, as well as minimizing the need for more complex appliances such as extraoral devices [1].

These devices offer several well-established advantages, including simple insertion and removal, cost-effectiveness, and versatility in facilitating a wide range of orthodontic tooth movements. Nevertheless, despite these benefits, instability, although relatively infrequent, and the occasional inability to maintain long-term osseous fixation remain significant limitations. Consequently, strategies aimed at improving the stability of these devices are of considerable clinical relevance throughout orthodontic treatment [1,2,3,4].

Orthodontic mini-implants are temporary anchorage devices whose biological behavior differs substantially from that of conventional dental implants. Unlike prosthetic implants, which rely on complete osseointegration to achieve long-term functional stability, orthodontic mini-implants are designed to remain stable through a combination of primary mechanical retention and subsequent peri-implant bone remodeling, without requiring true osseointegration. This biological characteristic allows their removal at the end of orthodontic treatment with minimal trauma to the surrounding bone [3,5].

Immediately after insertion, the placement of the mini-implant produces a controlled injury to the cortical and trabecular bone, initiating the physiological wound healing cascade. Bleeding at the implant–bone interface leads to the formation of a fibrin-rich blood clot, which acts as a temporary scaffold for the migration of inflammatory cells, mesenchymal stem cells, endothelial cells, and osteoprogenitor cells. Growth factors released by activated platelets, including platelet-derived growth factor (PDGF), transforming growth factor-β (TGF-β), and vascular endothelial growth factor (VEGF), stimulate angiogenesis and initiate the cascade of bone repair [5].

During the first days after insertion, osteoclast-mediated resorption removes areas of compressed and damaged bone created during implant placement. This transient resorptive phase results in a temporary reduction in primary mechanical stability. Simultaneously, osteoblasts begin depositing new osteoid matrix on the surrounding bone surfaces, which progressively mineralizes and is remodeled into mature lamellar bone. Rather than establishing a direct ankylotic bone-to-implant interface as observed in osseointegrated dental implants, this remodeling process creates a dynamic bone–implant interface capable of resisting orthodontic loading while preserving the temporary nature of the anchorage device [5,6].

Consequently, mini-implant stability is generally described as occurring in two biologically distinct phases. Primary stability is achieved immediately after insertion and depends almost exclusively on mechanical interlocking between the implant threads and the surrounding cortical bone. It is mainly influenced by cortical bone thickness, bone density, insertion torque, implant geometry, and surgical technique. As bone remodeling progresses, primary stability gradually decreases due to the resorption of compressed bone adjacent to the implant surface. At the same time, secondary stability progressively develops as newly formed bone matures and reorganizes around the implant. This transition period, which extends over the first several weeks after placement, represents the phase of greatest susceptibility to mini-implant failure because the reduction in mechanical stability precedes the establishment of sufficient biological stability [4,5,6,7,8].

It is estimated that the failure rate of orthodontic mini-implants is around 13.5%. However, this depends on the anatomical region of insertion. Mini-implants placed in the palatal region have a lower tendency to fail than those placed in the infrazygomatic region [2,9,10].

The region where the mini-implant is placed is important for stability. If it is placed close to the dental roots, less than 2 mm away, it may lead to the loss of the mini-implant. On the other hand, the insertion torque of the mini-implant is also important not only to prevent fractures of the mini-implant, but also to prevent microfractures of the bone around the mini-implant, thus leading to a decrease in primary stability and consequent loss of the mini-implant [10].

The adaptive response of the bone to orthodontic loads influences bone–implant contact, the maintenance of stability, and consequently, the success rate of temporary anchorage devices. Excessive alterations in bone remodeling, often associated with surgical microdamage or unfavorable bone conditions, can increase the risk of mobility and early failure of the mini-implant [11,12].

Photobiomodulation therapy using diode lasers has demonstrated promising results in enhancing the stability of orthodontic mini-implants, as well as accelerating orthodontic tooth movement, and has therefore attracted increasing research interest in recent years, however, there is still a large scientific gap regarding this topic [13,14,15,16,17,18,19,20,21].

The mechanisms underlying photobiomodulation have been extensively investigated. It is suggested that light absorption by intracellular chromophores, particularly cytochrome c oxidase, leads to increased adenosine triphosphate (ATP) production, modulation of reactive oxygen species, and activation of intracellular signaling pathways. These biological effects ultimately promote cellular proliferation and differentiation, enhance osteoblastic activity, modulate inflammatory responses, and accelerate tissue repair. In bone tissue, photobiomodulation has been associated with increased bone formation and improved quality of newly formed bone, suggesting a potential role in enhancing the secondary stability of orthodontic mini-implants [22].

The effectiveness of this therapy is based on a multifactorial modulation articulated across four interdependent biological domains. Firstly, at the level of blood vessels, PBM induces angiogenesis and improves local oxygen supply through the upregulation of growth factors. Simultaneously, it modulates the inflammatory response, promoting the phenotypic transition of macrophages to a reparative profile and coordinating the initial release of inflammatory interleukins essential for tooth movement, which in turn attenuates painful symptoms. Regarding the extracellular matrix, light stimulation accelerates the synthesis of collagen and periodontal fibers via activation of intracellular signaling pathways. Finally, at the level of mineralized tissues, photobiomodulation restores bone homeostatic balance, activating specific pathways that promote osteoclastogenesis on the compression side and osteogenesis on the tension side, demonstrating efficacy even in unfavorable metabolic scenarios [23,24].

Despite the growing number of studies on photobiomodulation in orthodontics, considerable heterogeneity remains in the protocols used. Wavelengths, fluence, application frequency, and treatment duration differ substantially between studies. Consequently, the most recent systematic reviews conclude that there is still no consensus regarding the ideal PBM protocol for accelerating orthodontic tooth movement [13,23,25,26,27,28,29].

The aim of this systematic review was to critically appraise and synthesize the available evidence from randomized controlled trials evaluating the effects of diode laser photobiomodulation on orthodontic mini-implant stability. The review was conducted to answer the following PICO question: In orthodontic patients requiring mini-implant anchorage (P), does diode laser photobiomodulation (I), compared with placebo or no photobiomodulation (C), improve mini-implant stability (O)?

## 2. Materials and Methods

The aim of this review was to evaluate the available scientific evidence on the effect of diode laser photobiomodulation on the stability of orthodontic mini-implants.

The review question and eligibility criteria were developed according to the Population, Intervention, Comparator, Outcome (PICO) framework. The review addressed the following question: “In orthodontic patients requiring temporary anchorage devices (orthodontic mini-implants), does diode laser photobiomodulation improve orthodontic mini-implant stability compared with placebo laser or no photobiomodulation?”

The PICO components were defined as follows: Population (P)—orthodontic patients requiring temporary anchorage devices (orthodontic mini-implants); Intervention (I)—diode laser photobiomodulation; Comparator (C)—placebo laser irradiation or no photobiomodulation; and Outcome (O)—orthodontic mini-implant stability assessed using objective methods, including resonance frequency analysis (RFA/ISQ) and Periotest (Medizintechnik Gulden e.K., Modautal, Germany) measurements.

A search of the PubMed/MEDLINE, EBSCO, Scopus and ScienceDirect databases was conducted in July 2026 using the following search terms in different combinations:

(“diode laser” OR “low-level laser therapy” OR photobiomodulation) AND (“orthodontic mini-implants” OR mini-screws OR “temporary anchorage devices” OR “mini-implant therapy”) AND (stability OR failure OR “survival rate”)

The search was limited to articles published in the last 10 years and written in English. Randomized controlled trials investigating the effects of diode laser photobiomodulation on the stability of orthodontic mini-implants were selected. Studies that evaluated other effects of photobiomodulation, publications that did not mention objective methods for assessing stability, and studies that did not directly address the topic of the review were excluded.

The publications were selected according to their relevance to the clinical question, according to previously defined inclusion and exclusion criteria:

- Randomized clinical trials (RCTs);
- Publications within the last 10 years;
- Use of diode laser for photobiomodulation;
- Objective assessment of orthodontic mini-implant stability.

The following exclusion criteria were defined:

- Studies that were not randomized clinical trials (RCTs);
- Publications prior to 2016;
- Use of photobiomodulation modalities other than diode laser;
- Assessment of mini-implant stability based on subjective or purely clinical methods.

Two reviewers independently screened titles and abstracts retrieved from the electronic databases according to the predefined eligibility criteria. Full-text articles considered potentially eligible were subsequently assessed independently. Any disagreements were resolved through discussion, and when consensus could not be reached, a third reviewer made the final decision. The study selection process was conducted according to the PRISMA 2020 guidelines [30] (Table S1).

The methodological quality of the included studies was independently assessed by two reviewers using the Cochrane Risk of Bias 2 (RoB 2) tool. Disagreements were resolved through discussion, and when necessary, by consultation with a third reviewer. The RoB 2 tool evaluates five domains of bias and provides an overall judgment of “low risk of bias”, “some concerns”, or “high risk of bias”.

The certainty of the evidence for the primary outcome was assessed using the Grading of Recommendations Assessment, Development and Evaluation (GRADE) approach. The certainty of evidence was classified as high, moderate, low, or very low after considering the risk of bias, inconsistency, indirectness, imprecision, and publication bias.

## 3. Results

**Figure 1:**
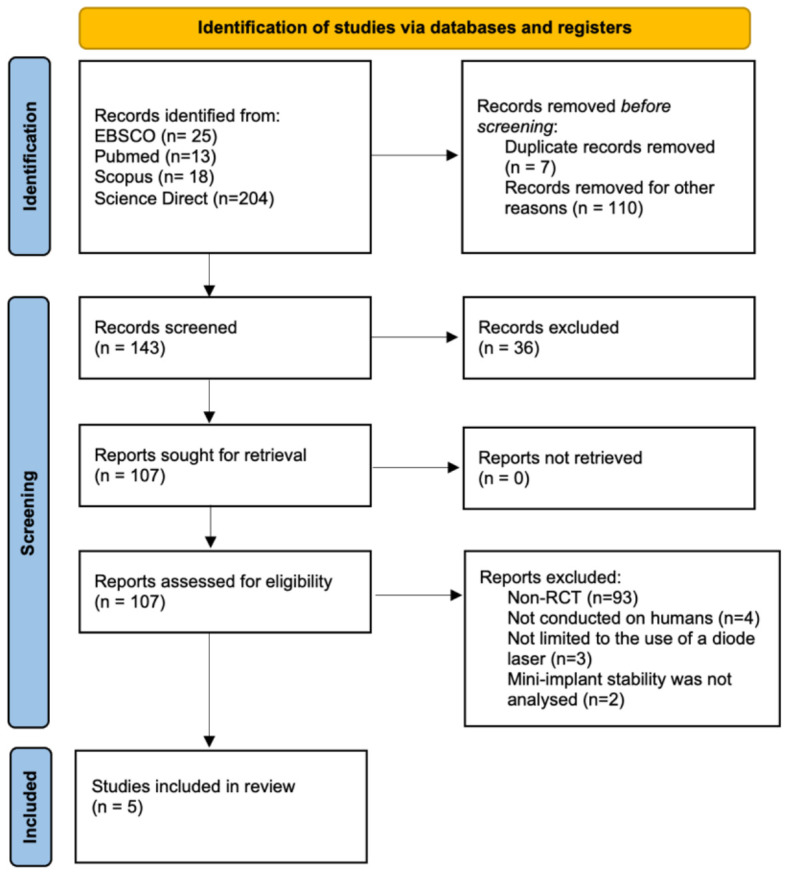
Flow diagram of the study selection process according to the PRISMA 2020 Statement.

The study selection process was conducted in accordance with the Preferred Reporting Items for Systematic Reviews and Meta-Analyses (PRISMA) 2020 Statement and is summarized in Figure 1.

The literature search identified 260 records from four electronic databases, including 204 from ScienceDirect, 25 from EBSCO, 18 from Scopus, and 13 from PubMed. After removing 7 duplicate records and 110 records before screening due to non-eligible publication types (e.g., editorials, book chapters, conference abstracts, and other non-scientific publications), 143 records remained for title and abstract screening.

Following the initial screening, 36 records were excluded because they did not meet the predefined eligibility criteria. The remaining 107 full-text articles were retrieved and assessed for eligibility, with no reports unavailable for retrieval.

Of the full-text articles assessed, 102 studies were excluded for the following reasons: 93 were not randomized controlled trials, 4 were not conducted in humans, 3 did not investigate photobiomodulation using a diode laser, and 2 did not objectively assess orthodontic mini-implant stability. Consequently, five randomized controlled trials met all predefined eligibility criteria and were included in the systematic review.

The main characteristics of the included studies are summarized in Table 1. A total of five randomized controlled trials, published between 2017 and 2023, met the predefined eligibility criteria and were included in the systematic review. Four studies employed a split-mouth randomized controlled design, whereas one was conducted as a split-mouth double-blind randomized controlled trial.

A total of 89 participants were initially enrolled across the five studies. Following the loss of one participant during follow-up in the study by Abohabib et al., data from 88 participants were included in the final analyses. Sample sizes ranged from 12 to 22 participants, with follow-up periods varying between 60 days and 12 weeks. All studies included orthodontic patients requiring maxillary mini-implants to provide skeletal anchorage during orthodontic treatment.

Mini-implants were inserted in the posterior maxilla, predominantly between the maxillary second premolar and first molar, although differences were observed regarding implant dimensions, insertion torque, insertion angulation, and orthodontic loading protocols. Three studies applied immediate orthodontic loading, one delayed loading by 14 days, and one did not apply orthodontic loading during the follow-up period. Mini-implant stability was objectively assessed using resonance frequency analysis (RFA) with the Osstell ISQ system (Osstell AB, Gothenburg, Sweden) or Periotest measurements obtained with the Periotest M device (Medizintechnik Gulden e.K., Modautal, Germany).

All included studies evaluated diode laser photobiomodulation; however, considerable heterogeneity was observed in the irradiation protocols. The wavelengths ranged from 635 to 940 nm, output power varied between 100 mW and 1.7 W, and the number of irradiation sessions ranged from 4 to 7. Additional variations were identified in irradiation time, fluence, total energy delivered, application mode (contact or non-contact), irradiation sites, and laser delivery protocols.

Overall, the included studies demonstrated heterogeneous findings regarding the effect of photobiomodulation on orthodontic mini-implant stability. Three studies reported statistically significant improvements in mini-implant stability following photobiomodulation, whereas two studies found no statistically significant differences compared with the control group. In addition, one study reported reduced peri-implant gingival inflammation following photobiomodulation, while no consistent effects were observed on postoperative pain or overall clinical success rates.

**Figure 2:**
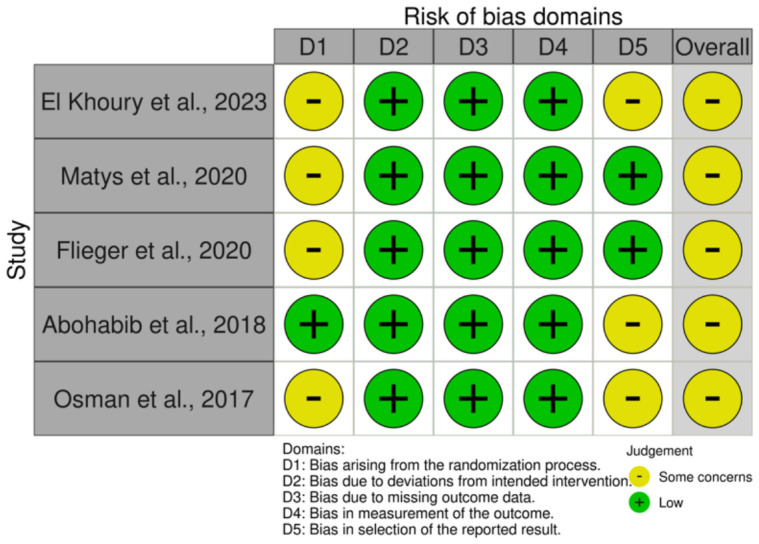
Risk of bias assessment of the included randomized controlled trials using the Cochrane Risk of Bias 2 (RoB 2) tool—traffic light plot [26,27,31,32,33].

**Figure 3:**
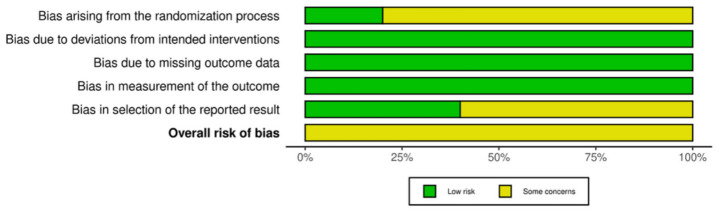
Summary of the risk-of-bias assessment across all included studies according to the Cochrane Risk of Bias 2 (RoB 2) tool—summary plot.

The methodological quality of the included studies was independently assessed by two reviewers using the Cochrane Risk of Bias 2 (RoB 2) tool. Any disagreements were resolved through discussion, and when necessary, by consultation with a third reviewer. The domain-specific assessments and overall risk-of-bias judgments are presented in Figure 2 and Figure 3.

All five included randomized controlled trials were judged as presenting some concerns regarding the overall risk of bias. Most domains were assessed as having a low risk of bias. However, some concerns were identified in several studies, primarily because of the insufficient reporting of allocation concealment and the absence of publicly available trial registration or prespecified statistical analysis plans. No study was classified as presenting an overall high risk of bias.

The certainty of the evidence for the primary outcome, orthodontic mini-implant stability, was assessed using the Grading of Recommendations Assessment, Development and Evaluation (GRADE) approach. The overall certainty of the evidence was judged as moderate. The certainty was downgraded by one level because of imprecision, reflecting the limited number of randomized controlled trials and the relatively small sample sizes included in the available studies. No serious concerns were identified regarding inconsistency, indirectness, or publication bias. The summary of findings is presented in Table 2.

The overall certainty of the evidence was judged as moderate according to the GRADE approach. Although all included randomized controlled trials received an overall judgment of some concerns in the RoB 2 assessment, these concerns were primarily attributable to the incomplete reporting of methodological details, particularly allocation concealment and the absence of publicly available trial registration or prespecified statistical analysis plans, rather than evidence of substantial methodological shortcomings likely to influence the estimated treatment effect. Consequently, no downgrade for risk of bias was considered appropriate. Instead, the certainty of the evidence was downgraded solely because of serious imprecision resulting from the limited number of available randomized controlled trials and their relatively small sample sizes. Therefore, while the current evidence suggests that diode laser photobiomodulation may improve orthodontic mini-implant stability, additional adequately powered randomized controlled trials are required to increase confidence in the estimated effect.

## 4. Discussion

The present systematic review aimed to evaluate the available evidence regarding the effects of diode laser photobiomodulation (PBM) on the stability of orthodontic mini-implants. Overall, the findings suggest that PBM may enhance mini-implant stability. Nevertheless, the available evidence remains heterogeneous, and the current data are insufficient to support the establishment of a standardized clinical protocol. The variability observed among the included studies indicates that the biological response to PBM is likely influenced by multiple interacting factors, including irradiation parameters, implant-related characteristics, orthodontic loading protocols, and methods used to assess implant stability.

Although three of the five included randomized controlled trials demonstrated statistically significant improvements in mini-implant stability following PBM, two studies reported no significant differences compared with placebo or control interventions. These apparently conflicting findings should not necessarily be interpreted as contradictory evidence regarding the efficacy of PBM. Instead, they likely reflect the substantial methodological and clinical heterogeneity observed across the available studies. Consequently, interpretation of the current evidence requires consideration not only of the statistical significance but also of the biological mechanisms underlying photobiomodulation and the methodological differences among the published clinical trials.

The most prominent source of heterogeneity identified in the present review was the considerable variation in laser irradiation protocols. Wavelengths ranged from 635 to 940 nm, output power varied between 100 mW and 1.7 W, and important differences were identified regarding fluence, irradiation time, total delivered energy, number of irradiation sessions, application mode, irradiation sites, and treatment schedules. Such variability has considerable biological implications because PBM does not represent a single therapeutic intervention but rather a group of treatments whose biological effects are highly dependent on irradiation parameters.

Photobiomodulation is primarily mediated through the absorption of photons by intracellular chromophores, particularly cytochrome c oxidase, the terminal enzyme of the mitochondrial respiratory chain. Photon absorption results in increased mitochondrial electron transport, enhanced ATP synthesis, transient modulation of reactive oxygen species, nitric oxide release, and activation of multiple intracellular signaling pathways involved in cell proliferation, differentiation, and tissue repair. In bone tissue, these mechanisms have been associated with increased osteoblastic proliferation, enhanced collagen synthesis, stimulation of angiogenesis, modulation of inflammatory cytokines, and accelerated bone remodeling. Collectively, these biological responses provide a plausible explanation for the improved peri-implant healing and increased mini-implant stability observed in several of the included studies [15,22,34].

It is known that different wavelengths trigger distinct cellular mechanisms: the application of a laser with a wavelength between 600 and 800 nm is absorbed by the cytochrome c oxidase enzyme, located in the mitochondrial respiratory chain, activating the enzyme through nitric oxide, originating a proton gradient, thus increasing the calcium and ATP levels. On the other hand, the application of a laser with a wavelength between 810 and 1064 nm increases the calcium levels through the activation of photosensitive ion channels. Although the cellular mechanisms are distinct, both lead to cell differentiation, proliferation, and migration [22].

The wavelengths used in the studies analyzed for this review ranged from 635 to 940 nm. Thus, three studies used lasers with wavelengths shorter than 810 nm, leading to the assumption that in two of them, ATP production through the activation of the cytochrome c oxidase enzyme by nitric oxide is also effective, as is the case with longer wavelengths through the activation of photosensitive calcium channels. However, no studies comparing the effectiveness of the two mechanisms through different wavelengths with respect to the stability of orthodontic mini-implants were found in the literature.

The biological response induced by PBM is strongly influenced by irradiation parameters. Experimental evidence consistently supports the existence of a biphasic dose–response relationship, commonly described by the Arndt–Schulz law, whereby insufficient irradiation fails to elicit an adequate biological response, whereas excessive energy delivery may reduce or even inhibit cellular activity. Consequently, apparently contradictory findings between clinical studies may simply reflect differences in laser protocols rather than true differences in treatment efficacy. This concept is particularly important when interpreting the present review, as none of the included studies employed identical irradiation parameters [35].

Another important aspect concerns the wavelength of the diode lasers used. The studies included in this review employed wavelengths ranging from 635 to 940 nm, corresponding to the optical window in which light penetration through biological tissues is maximized. Nevertheless, different wavelengths exhibit distinct optical properties and tissue penetration depths. Lower wavelengths, such as 635 nm, are characterized by greater absorption by superficial tissues, including hemoglobin and melanin, resulting in relatively limited penetration into deeper bone structures. Otherwise, wavelengths within the near-infrared spectrum, particularly 808–940 nm, exhibit reduced absorption by superficial chromophores and therefore penetrate more deeply into biological tissues, allowing a greater proportion of the delivered energy to reach the peri-implant bone. These differences may partly explain the variability observed among clinical studies despite apparently similar energy densities [25,35].

Importantly, wavelength alone is unlikely to determine clinical outcomes. Tissue penetration also depends on several additional variables, including irradiance, beam profile, exposure time, energy density, application angle, contact or non-contact mode, and the optical characteristics of the irradiated tissues. Consequently, two protocols employing identical wavelengths may still produce substantially different biological effects if other irradiation parameters differ. Likewise, similar clinical outcomes may occasionally be obtained using different wavelengths when the overall energy delivered to the target tissues is comparable. This complexity further illustrates why direct comparison between currently available clinical trials remains challenging.

The lack of protocol standardization identified in the present review is consistent with previous literature investigating PBM in orthodontics and represents one of the major obstacles preventing the development of evidence-based clinical recommendations. Although numerous experimental investigations have demonstrated favorable cellular responses following PBM, there is currently no consensus regarding the optimal wavelength, power output, fluence, irradiation frequency, treatment duration or cumulative energy required to maximize peri-implant bone healing. Consequently, clinicians remain unable to identify the protocol most likely to produce predictable improvements in orthodontic mini-implant stability [24,25,28,29].

The temporal pattern of the reported clinical outcomes also deserves consideration. Interestingly, the beneficial effects observed in the positive studies generally became evident several weeks after mini-implant placement rather than immediately following insertion. This finding closely coincides with the transition from primary mechanical stability to secondary biological stability, suggesting that PBM may exert its principal effects during peri-implant bone healing rather than during the initial mechanical retention phase. Primary stability depends predominantly on cortical bone engagement, implant geometry, insertion torque, and bone quality, whereas secondary stability develops progressively through bone remodeling and repair around the implant interface. Therefore, it is biologically plausible that PBM would primarily influence secondary stability by accelerating cellular and molecular events associated with bone healing. This hypothesis is further supported by the timing of mini-implant failures reported in the orthodontic literature [2,3,4].

In addition to irradiation protocols, several other methodological differences may have influenced the reported outcomes. Orthodontic loading protocols varied considerably among the included studies. While most investigations applied immediate loading, one delayed orthodontic loading by 14 days and another did not apply orthodontic loading during the observation period. Mechanical loading directly influences peri-implant bone remodeling and therefore represents an important confounding factor when evaluating the biological effects of PBM. Similarly, implant dimensions, insertion torque, insertion angulation, cortical bone thickness, and surgical techniques differed among studies and are all recognized determinants of primary stability that may subsequently influence biological healing [36,37].

Methodological heterogeneity was also observed in the assessment of implant stability. The included studies employed either resonance frequency analysis (RFA) using the Osstell ISQ system (Osstell AB, Gothenburg, Sweden) or Periotest measurements obtained with the Periotest M device (Medizintechnik Gulden e.K., Modautal, Germany). Although both techniques are validated and widely accepted for evaluating implant stability, they measure different biomechanical characteristics and therefore cannot be considered directly interchangeable. Consequently, the comparison of absolute stability values between studies should be interpreted with caution, particularly when attempting to compare the magnitude of treatment effects across different measurement systems.

The findings of the present review are generally consistent with those reported in previous systematic reviews evaluating the effects of photobiomodulation on orthodontic mini-implant stability, although important differences in eligibility criteria and methodological approaches should be considered. Zhang et al. included seven clinical studies comprising randomized and non-randomized controlled trials, as well as investigations using both diode lasers and light-emitting diode (LED) devices. Their meta-analysis, based on only two studies, demonstrated a significant improvement in mini-implant stability after approximately 60 days, whereas no significant differences were observed at earlier time points. The authors also suggested that protocols employing higher energy densities may induce earlier biological responses than those using lower irradiation doses. However, this conclusion was derived from indirect comparisons among heterogeneous studies rather than from randomized comparisons between different laser parameters, limiting the strength of this observation [14].

Similarly, Mohan et al. performed a systematic review and meta-analysis including six randomized controlled trials investigating photobiomodulation and orthodontic mini-implant stability. Their quantitative synthesis demonstrated significantly lower Periotest values in the photobiomodulation groups after both 30 and 60 days, suggesting improved implant stability during the healing period. Nevertheless, the authors emphasized that the available evidence should be interpreted cautiously because of the limited number of studies, relatively small sample sizes, and substantial heterogeneity in irradiation protocols. These conclusions closely align with those of the present review, reinforcing the hypothesis that photobiomodulation may positively influence peri-implant bone healing while simultaneously highlighting the current limitations of the available evidence [38].

Despite these similarities, the conclusions of the present review are slightly more conservative than those of previous systematic reviews. This difference may largely be explained by the stricter eligibility criteria adopted. Only randomized controlled trials conducted in humans were included, photobiomodulation was restricted exclusively to diode lasers, and studies were required to evaluate mini-implant stability using objective measurement methods. Consequently, studies employing LED devices, non-randomized designs, subjective outcome assessment, or interventions not directly related to diode laser photobiomodulation were excluded. Furthermore, the present review incorporated the more recent randomized controlled trial by El Khoury et al., which reported no statistically significant differences between photobiomodulation and the placebo throughout the observation period. The inclusion of this more recent evidence contributes to a more balanced interpretation of the currently available literature and suggests that the beneficial effects of photobiomodulation may be less consistent than previously suggested.

Comparison with the broader implantology literature also provides valuable insights into the biological effects of photobiomodulation. Qu et al. evaluated the influence of photobiomodulation on conventional dental implants and reported improved implant stability approximately ten days after implant placement together with reduced marginal bone loss after six months. However, no consistent improvements were observed at later follow-up periods or in several secondary clinical outcomes, including postoperative pain, peri-implant clinical parameters, and bone mineral density. These findings indicate that photobiomodulation may primarily influence the early stages of bone healing while exerting a less pronounced effect on long-term implant outcomes [39].

Nevertheless, caution should be exercised when extrapolating findings from conventional implantology to orthodontic mini-implants. Unlike dental implants, which are specifically designed to achieve long-term osseointegration, orthodontic mini-implants function as temporary anchorage devices and rely predominantly on mechanical retention rather than complete osseointegration. Their smaller dimensions, minimally invasive placement, immediate or early orthodontic loading, and temporary clinical function create a biological environment that differs substantially from that surrounding conventional dental implants. Consequently, although the biological mechanisms underlying photobiomodulation are likely to be similar, the clinical response may differ because of the distinct biomechanical requirements of orthodontic mini-implants.

The methodological quality of the included studies should also be considered when interpreting the findings of the present review. According to the Cochrane Risk of Bias 2 (RoB 2) tool, all included randomized controlled trials received an overall judgment of some concerns. Importantly, these judgments primarily reflected incomplete methodological reporting rather than clear evidence of substantial bias. The principal concerns related to insufficient information regarding allocation concealment, and in several studies, the absence of publicly available trial registration or pre-specified statistical analysis plans. Nevertheless, all included studies employed randomized allocation, objective outcome assessment, and complete or nearly complete follow-up data, reducing the likelihood that these limitations substantially affected the reported findings.

The certainty of the evidence was assessed using the GRADE approach and was judged to be moderate for the primary outcome of orthodontic mini-implant stability. The certainty of evidence was downgraded by one level because of serious imprecision resulting from the relatively small sample sizes and the limited number of available randomized controlled trials. No serious concerns were identified regarding indirectness or publication bias. Although some variability was observed among the included studies, this heterogeneity was considered largely attributable to differences in clinical protocols rather than to inconsistent biological effects. Consequently, the overall certainty of evidence suggests that photobiomodulation may improve orthodontic mini-implant stability, although future well-designed randomized clinical trials are likely to further refine the estimated treatment effect.

Several limitations of the present systematic review should be acknowledged. First, the review protocol was not prospectively registered in the International Prospective Register of Systematic Reviews (PROSPERO). Therefore, the absence of prospective registration represents an important methodological limitation and should be considered when interpreting the findings.

Nevertheless, several methodological safeguards were implemented to minimize potential bias. The review was conducted and reported in accordance with the PRISMA 2020 Statement, the research question and eligibility criteria were established a priori using the PICO framework, and all stages of study selection, data extraction, risk-of-bias assessment, and certainty-of-evidence assessment were performed independently by two reviewers. Any disagreements were resolved through discussion, and whenever consensus could not be achieved, by consultation with a third reviewer with greater methodological experience. These procedures substantially strengthened the transparency, reproducibility, and methodological robustness of the review despite the absence of prospective registration [34].

Additional limitations relate to the relatively small number of eligible randomized controlled trials and the limited overall sample size currently available. Furthermore, considerable clinical and methodological heterogeneity was identified among the included studies, particularly regarding laser wavelength, irradiation parameters, treatment schedules, orthodontic loading protocols, follow-up periods, mini-implant characteristics, and methods used to assess stability. These differences prevented quantitative synthesis through meta-analysis and limited a direct comparison of treatment effects across studies.

Despite these limitations, the present review possesses several important strengths. This is one of the few systematic reviews focusing exclusively on human randomized controlled trials investigating diode laser photobiomodulation for orthodontic mini-implant stability. By excluding non-randomized studies, studies involving alternative light sources, and investigations lacking objective stability assessment, the present review provides a more homogeneous synthesis of the highest level of currently available clinical evidence. Furthermore, the methodological quality of the included studies and the certainty of the available evidence were systematically evaluated using internationally recognized assessment tools (RoB 2 and GRADE), thereby enhancing the reliability of the review findings.

From a clinical perspective, diode laser photobiomodulation appears to represent a promising adjunctive strategy for improving orthodontic mini-implant stability, particularly during the early healing phase in which secondary biological stability is established. However, the available evidence remains insufficient to support routine clinical implementation or to recommend a specific irradiation protocol. At present, no consensus exists regarding the optimal wavelength, power output, energy density, irradiation frequency, treatment duration, or cumulative energy required to maximize clinical efficacy. Consequently, clinicians should interpret the current evidence cautiously and recognize that protocol selection remains largely empirical.

Future randomized controlled trials should prioritize the development of standardized photobiomodulation protocols while providing comprehensive reporting of all irradiation parameters in accordance with current photobiomodulation reporting recommendations. Larger multicenter studies with adequate statistical power, longer follow-up periods, and standardized methods for assessing mini-implant stability are also required. In addition to stability measurements, future investigations should evaluate clinically meaningful outcomes, including mini-implant survival, failure rates, time to orthodontic loading, patient-reported outcomes, treatment duration, and cost-effectiveness. Such improvements would facilitate comparison among studies, enable robust quantitative syntheses, and ultimately strengthen the certainty of the available evidence.

Overall, the present systematic review provides a comprehensive synthesis of the highest-quality clinical evidence currently available regarding the use of diode laser photobiomodulation as an adjunctive therapy for improving orthodontic mini-implant stability. Although the available evidence is encouraging and supports a potential beneficial effect of photobiomodulation, the absence of standardized irradiation protocols, the limited number of randomized controlled trials, and the uncertainty regarding the clinical magnitude of the observed effects indicate that further high-quality clinical research is required before definitive evidence-based recommendations can be established.

## 5. Conclusions

Within the limitations of the currently available evidence, diode laser photobiomodulation may improve the stability of orthodontic mini-implants, particularly during the early healing period associated with the establishment of secondary biological stability. However, the available evidence remains limited by the small number of randomized controlled trials, relatively small sample sizes, and substantial heterogeneity in laser parameters, irradiation protocols, orthodontic loading strategies, and methods used to assess implant stability.

Although most of the included studies reported favorable effects of photobiomodulation, the current evidence is insufficient to establish a standardized clinical protocol or to determine the optimal wavelength, energy density, irradiation schedule, or total delivered energy required to maximize treatment efficacy. Furthermore, the clinical significance of the observed improvements remains uncertain, as evidence demonstrating meaningful reductions in mini-implant failure rates or improvements in long-term clinical outcomes is still lacking.

Future well-designed, adequately powered randomized controlled trials employing standardized photobiomodulation protocols and uniform outcome assessment methods are required to strengthen the certainty of the available evidence and establish evidence-based clinical recommendations for the use of diode laser photobiomodulation in orthodontic mini-implant therapy.

## Figures and Tables

**Table 1:** Summary of the analysis of the selected articles.

Study	Study and Participant Characteristics	Mini-Implant & Orthodontic Loading	PBM Protocol	Outcomes
El Khoury et al., 2023 [31]	Split-mouth, double-blind randomized controlled trial. Twenty patients (8 males, 12 females; mean age: 22.9 ± 4.78 years) with skeletal and dental Class II malocclusion requiring bilateral maxillary mini-implant anchorage for canine distalization. Follow-up: 12 weeks (3 months).	Forty self-drilling mini-implants (M.O.S.A.S., Dewimed, Germany), 1.6 × 8 mm, inserted using 40 N·cm insertion torque. Mini-implants were placed at 90°, 1 mm below the mucogingival junction, between the first and second premolars or between the second premolar and first molar. Cortical bone thickness was not reported. Orthodontic loading was immediate, using identical bilateral forces verified with a dynamometer; the exact force magnitude was not reported. Stability was assessed by resonance frequency analysis (Osstell ISQ).	Device: Picasso Lite^®^ (AMD Lasers, West Jordan, UT, USA). Laser: GaAlAs diode laser. Wavelength: 810 nm. Output power: 1 W. Irradiance: 1.2 W/cm^2^. Spot size: 0.8 cm^2^. Energy per session: 50 J. Fluence: 8.3 J/cm^2^. Optical fiber: 400 μm non-initiated tip. Emission mode: Continuous wave (CW). Application mode: Non-contact, sweeping movement, 22 mm from the mini-implants, movement speed 1 mm/s. Irradiation points: Not point-specific (continuous sweeping over the mini-implant). Application time: 50 s/session. Sessions: Baseline, days 2 and 4, and weeks 3, 6, 9 and 12 (7 sessions). Total delivered energy: 350 J. Sham treatment: Laser switched off (0 W; 0 J/cm^2^) with a 632 nm red guide beam to maintain blinding.	Mini-implant stability (lower, upper, distal and global ISQ) decreased significantly over time in both the laser and placebo groups (*p* < 0.001). No statistically significant differences were observed between irradiated and placebo mini-implants for lower (*p* = 0.920), upper (*p* = 0.955), distal (*p* = 0.689) or global ISQ (*p* = 0.842). The authors concluded that 810-nm PBM did not significantly improve secondary stability of orthodontic mini-implants over a 3-month period, although they suggested that protocol optimization and more standardized PBM parameters should be investigated in future studies.
Matys et al., 2020 [27]	Split-mouth randomized controlled trial. Twenty-two patients (14 females, 8 males; mean age: 31.7 ± 9.7 years) with skeletal Class II malocclusion requiring maxillary distalization. Follow-up: 60 days.	Grade V titanium mini-implants (RMO, Franklin, IN, USA), 1.4 × 10 mm, inserted between the maxillary second premolar and first molar, 2 mm below the mucogingival junction, using a self-drilling technique without cortical decortication. No orthodontic loading was applied during the study period. Implant stability was assessed using the Periotest.	Device: SmartM PRO (Lasotronix, Piaseczno, Poland). Laser: 808 nm diode laser. Output power: 100 mW. Irradiance: 200 mW/cm^2^. Spot size: 0.5 cm^2^. Energy/point: 4 J. Fluence: 8 J/cm^2^. Mode: Continuous wave (CW). Application: contact, two irradiation points (buccal and palatal), 40 s/point (80 s/session). Sessions: immediately after insertion and at 3, 6, 9, 12, 15 and 30 days (7 sessions). Total energy: 56 J. Control: contralateral non-irradiated mini-implant.	No significant differences in stability at baseline or after 3, 6, 9, 12 and 15 days (*p* > 0.05). PBM significantly increased secondary mini-implant stability after 30 days (PTV: 6.32 ± 3.62 vs. 11.34 ± 5.76; *p* = 0.004) and 60 days (PTV: 6.55 ± 4.66 vs. 10.95 ± 4.77; *p* = 0.009). No significant difference in postoperative pain (*p* = 0.499). One mini-implant failed in the control group, whereas no failures occurred in the laser group. Authors concluded that PBM enhanced secondary mini-implant stability.
Flieger et al., 2020 [26]	Randomized clinical split-mouth trial. Twenty patients (13 women, 7 men; mean age 32.5 ± 6.1 years) with Class II malocclusion requiring bilateral maxillary mini-implants for distalization. Forty mini-implants were evaluated (20 irradiated; 20 controls). Follow-up: 60 days.	Forty self-drilling titanium alloy (Grade V) mini-implants (RMO, Franklin, IN, USA), 1.4 mm × 10 mm, were inserted between the maxillary second premolar and first molar, 2 mm below the mucogingival junction, after soft-tissue removal with a ceramic bur and without bone decortication. All procedures were performed by the same surgeon. The orthodontic appliance consisted of 0.022-inch MBT fixed appliances. The article does not specify the timing or magnitude of orthodontic loading, nor cortical bone thickness or insertion torque. Implant stability was assessed using the Periotest device (PTV). Lower PTV values indicate greater stability.	Device: SmartM^®^ (Lasotronix, Piaseczno, Poland). Laser: Red diode laser. Wavelength: 635 nm. Output power: 100 mW. Spot size: 0.5024 cm^2^ (8 mm handpiece). Power density: 199.04 mW/cm^2^. Emission mode: Continuous wave (CW). Application mode: Contact. Energy: 10 J per point (20 J/cm^2^). Application time: 100 s per point. Irradiation sites: 2 points (buccal and palatal aspects of each mini-implant). Energy per session: 20 J. Sessions: Immediately after insertion and at 3, 6, 9, 12, 15 and 30 days (7 sessions). Total delivered energy: 140 J. The contralateral side served as the non-irradiated control	PBM significantly improved secondary stability of orthodontic mini-implants. The irradiated group showed significantly lower (better) Periotest values than controls after 3 days (*p* < 0.0001), 30 days (*p* = 0.0003) and 60 days (*p* < 0.0001). No statistically significant differences were observed at baseline, 6, 9, 12 or 15 days. No significant differences in postoperative pain were found between groups (*p* = 0.3665). No mini-implant failures occurred during the 60-day follow-up, resulting in a 100% survival rate in both groups. The authors concluded that 635-nm photobiomodulation enhances secondary stability of orthodontic mini-implants without affecting postoperative pain.
Abohabib et al., 2018 [32]	Split-mouth randomized controlled clinical trial. Fifteen patients (mean age 20.9 ± 3.4 years) requiring extraction of the maxillary first premolars followed by bilateral canine retraction. One participant was lost to follow-up, leaving 14 patients (28 mini-implants; 14 laser, 14 control) for analysis. Follow-up: 10 weeks.	AbsoAnchor^®^ self-drilling orthodontic mini-implants (Dentos, Daegu, Republic of Korea), 1.5 × 8 mm, inserted bilaterally between the maxillary second premolar and first molar, 1 mm coronal to the mucogingival junction, almost perpendicular to the alveolar bone. Insertion torque: 7 N·cm. Cortical bone thickness was not reported. Mini-implants were immediately loaded with 150 g of force using nickel–titanium closed-coil springs for canine retraction. Stability was measured using resonance frequency analysis (Osstell ISQ/SmartPeg)	Device: Biolase Epic 10 Console^®^ diode laser (Irvine, CA, USA). Laser: InGaAsP diode laser. Wavelength: 940 nm. Output power: 1.7 W. Emission mode: Continuous wave (CW). Energy density (fluence): 36 J/cm^2^. Application mode: Non-contact, perpendicular to the mini-implant using a tooth-whitening handpiece. Application time: 60 s per session. Sessions: Days 0, 7, 14 and 21 after mini-implant placement (4 sessions). Total fluence: 144 J/cm^2^. Energy per point, spot size, irradiance, and total energy (J) were not reported. Sham treatment: identical handpiece positioning without laser activation.	The overall clinical success rate was 78.5%, with no difference between laser and control groups (three failures in each group). Resonance frequency values were not significantly different from baseline to week 2 (*p* > 0.05). From weeks 3 to 10, the laser group demonstrated significantly higher resonance frequency values than controls (week 3: *p* = 0.032; week 4: *p* = 0.047; week 6: *p* = 0.016; week 8: *p* = 0.037; week 10: *p* = 0.040). Despite these improvements in resonance frequency, no statistically significant improvement in clinical mini-implant stability or success rate was observed. The authors concluded that 940-nm low-intensity laser therapy cannot be recommended as a clinically useful adjunct for improving orthodontic mini-implant stability during canine retraction.
Osman et al., 2017 [33]	Split-mouth randomized controlled clinical trial. Twelve patients (6 males, 6 females; mean age: 18 years) requiring bilateral extraction of the maxillary first premolars and absolute anchorage for canine retraction. All participants completed the 2-month follow-up.	Twenty-four AbsoAnchor^®^ mini-implants (Dentos, Daegu, Republic of Korea), inserted in the buccal alveolar bone between the maxillary second premolar and first molar at approximately 60° to the alveolar bone. Mini-implant dimensions, insertion torque and cortical bone thickness were not reported. Orthodontic loading was delayed for 14 days after placement using 150 g NiTi closed-coil springs for canine retraction. Stability was assessed using the Periotest^®^ device.	Device: Biolase Technology Inc. diode laser. Laser: diode laser. Wavelength: 910 nm. Output power: 0.7 W (700 mW). Emission mode: Continuous wave (CW). Application mode: Non-contact over the soft tissue surrounding the mini-implant. Application time: 60 s/session. Sessions: Immediately after insertion and every 72 h during the first 14 days (4 sessions). Spot size, irradiance, fluence, energy per point and total energy delivered: not reported. Sham treatment: identical application with the laser switched off on the contralateral side.	Periotest values were consistently lower (greater stability) in the laser group than in the control group throughout the study; however, none of the differences reached statistical significance (baseline: *p* = 0.679; 7 days: *p* = 0.480; 14 days: *p* = 0.381; 21 days: *p* = 0.451; 1 month: *p* = 0.225; 2 months: *p* = 0.159). Comparison between baseline and 2 months also showed no significant change in either the laser (*p* = 0.648) or control group (*p* = 0.341). In contrast, peri-implant gingival inflammation was markedly reduced in the laser group, with no inflammatory signs throughout follow-up, whereas the control group developed mild to moderate gingival inflammation in several patients after 1–2 months. The authors concluded that LLLT may improve mini-implant stability and peri-implant soft tissue health, although the improvement in stability was not statistically significant, recommending further long-term studies with larger sample sizes.

**Table 2:** Summary of findings and certainty of the evidence according to the GRADE approach. Abbreviations: RCTs, randomized controlled trials. GRADE certainty ratings: ⨁⨁⨁⨁, high certainty; ⨁⨁⨁◯, moderate certainty; ⨁⨁◯◯, low certainty; ⨁◯◯◯, very low certainty.

Outcome	Nº of Studies	Participants	Risk of Bias	Inconsistency	Indirectness	Imprecision	Publication Bias	Overall Certainty
Orthodontic mini-implant stability	5 RCTs	89 participants	Not serious	Not serious	Not serious	Serious	Undetected	⨁⨁⨁◯ Moderate

## Data Availability

No new data were created or analyzed in this study. Data sharing is not applicable to this article.

## Supplementary Materials

The following supporting information can be downloaded at: https://www.mdpi.com/article/10.3390/jcm15145628/s1, Table S1: PRISMA 2020 Checklist.
